# Developing a Trauma-Informed Social Media Campaign to Disseminate Endometriosis-Specific Qualitative Art-Based Research Findings: Tutorial

**DOI:** 10.2196/83491

**Published:** 2026-01-27

**Authors:** Kerry Marshall, Hargun Dhillon, A Fuchsia Howard, Heather Noga, Grace J Yang, William Zhu, Jessica Sutherland, Sarah Lett, Anna Leonova, Paul J Yong, Natasha L Orr

**Affiliations:** 1 School of Nursing, University of British Columbia, Gateway Health Building, 5955 University Blvd., Vancouver, BC, V6T 1Z1, Canada, 1 604-822-4372; 2 Women’s Health Research Institute, BC Women’s Hospital & Health Centre, Vancouver, BC, Canada; 3 Department of Obstetrics and Gynecology, Faculty of Medicine, University of British Columbia, Vancouver, BC, Canada; 4 Patient Research Advisory Board, Endometriosis and Pelvic Pain Lab, University of British Columbia, Vancouver, BC, Canada

**Keywords:** trauma-informed approach, social media, knowledge translation, endometriosis, information dissemination, content creation

## Abstract

Trauma-informed approaches can promote the creation of systems that prioritize safety and empowerment to improve patient well-being. These approaches are especially important in sexual and reproductive health care, where patients are often asked to disclose sensitive and personal information. This disclosure is particularly relevant in the context of endometriosis, a condition that affects 10% of reproductive-aged women and causes debilitating pelvic pain. Our team led a trauma-informed social media campaign to raise awareness and improve the understanding of endometriosis by sharing research findings from a photovoice study focusing on Asian women’s experiences of endometriosis during the COVID-19 pandemic in Canada (*EndoPhoto Study*). In this paper, we describe how we adapted and applied trauma-informed approaches to the development and implementation of the social media campaign. To do this, we followed five adapted trauma-informed principles: (1) support and collaboration, (2) trustworthiness and transparency, (3) safety, (4) empowerment and voice, and (5) cultural and gender sensitivity, and four steps: (1) frame the campaign, (2) create content and manage the campaign, (3) measure campaign impact, and (4) conduct postcampaign reflections. We co-designed this campaign with patient partners having lived experience of endometriosis to facilitate support and collaboration. Additionally, we shared details about the funders of this study to increase trust and transparency, moderated comments and deidentified images to promote participant safety, chose safer platforms to enhance empowerment and voice, avoided stereotypes, and shared authentic experiences of Asian women with endometriosis to support cultural and gender sensitivity. The campaign launched on Instagram and Pinterest in March 2025 to coincide with Endometriosis Awareness Month. The social media campaign received 8,540,528 total impressions over the course of the month and had engagement rates of 6.23% and 1.4% on Instagram and Pinterest, respectively.

## Background and Rationale

### Overview

Endometriosis is a chronic inflammatory condition characterized by the presence of endometrial-like tissue outside the uterus [1]. The symptoms may vary, but they often include severe pelvic pain, painful periods, painful sexual intercourse, and infertility [2]. Despite affecting approximately 10% of reproductive-aged women and girls, and an unmeasured number of gender diverse people, endometriosis remains significantly underdiagnosed and misunderstood [1,3]. Although diagnostic delays average 5 years in Canada, some individuals have reported a formal diagnosis taking up to 20 years [3,4]. The invisibility of symptoms, stigma surrounding sexual and menstrual health, and dismissal of women’s pain all contribute to misinformation and present barriers to timely diagnosis and treatment [3,5,6]. Ultimately, these aspects all affect the mental and physical health of those with endometriosis. Furthermore, racialized populations may experience additional barriers to endometriosis diagnosis and care [7]. For instance, one study found that East and/or Southeast Asian women were 8 times more likely than their White counterparts to experience severe disease before being referred to more specialized care [8].

Globally, the COVID-19 pandemic further exacerbated the gaps in endometriosis care as it upended the health care system, causing resource redirection toward patients with COVID-19 and interrupting the continuity of care for patients with chronic conditions like endometriosis [9,10]. In Canada, appointments and surgeries for people with endometriosis were postponed or canceled as hospitals became overwhelmed and health care providers transitioned to virtual environments [11]. Concurrently, mandatory self-isolation measures dramatically altered people’s levels of social support, contributing to worsening psychological symptoms such as depression and anxiety [12]. Additionally, the COVID-19 pandemic was marked by a global rise in anti-Asian sentiment, with people of Asian descent reporting increasing episodes of violence and feelings of vulnerability to discrimination [13].

Given these compounding factors, our team conducted a study—the *EndoPhoto Study—*with 22 South, East, and/or Southeast Asian cisgender women with endometriosis in Canada to better understand the experiences of people in these communities during the COVID-19 pandemic. This study used photovoice, an art-based methodology that provides opportunities to use photos to share experiences and emotions related to stigmatized or hidden conditions [14]. Results from the *EndoPhoto Study* are published elsewhere [15,16] and highlight several key themes. These themes include the ways in which the pandemic exacerbated feelings of isolation and created additional challenges in accessing health care for those living with endometriosis. Participants also built resilience during the pandemic by accepting social support from peers, advocating for themselves in health care interactions, and taking empowering actions to self-manage their conditions. More details on the methodology and data analysis of the *EndoPhoto Study* are available in other studies [15,16]. The *EndoPhoto Study* was approved by the University of British Columbia Children’s and Women’s Research Ethics Board (reference number: H22-02390).

**Figure 1.**
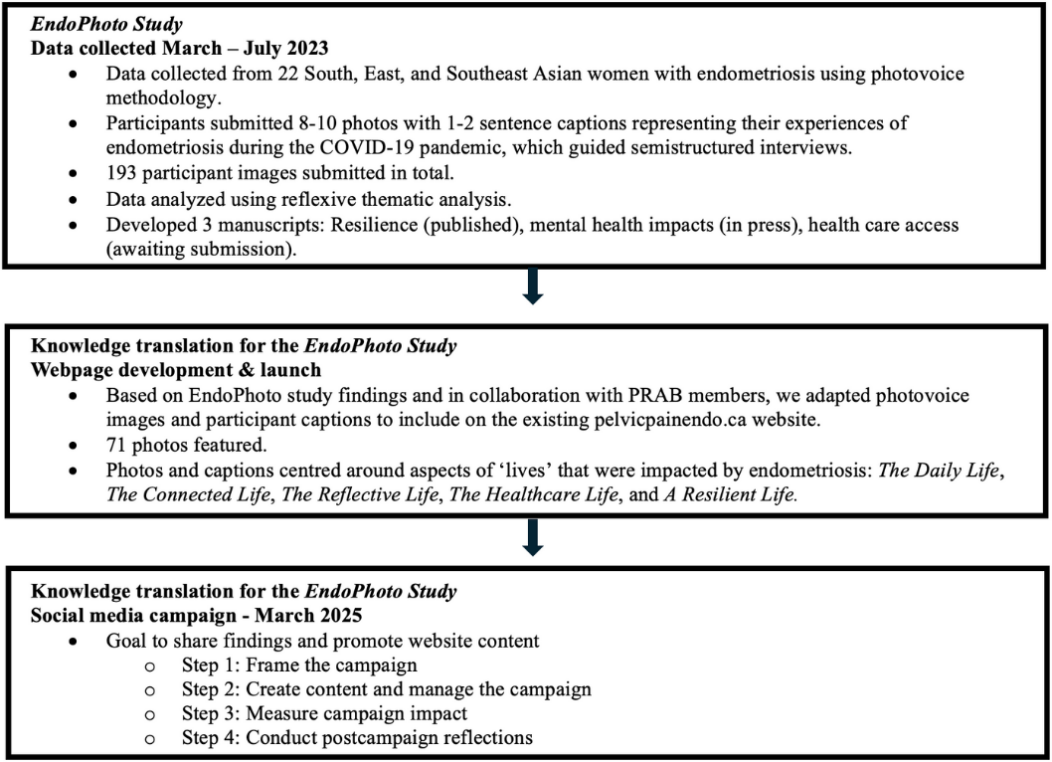
Project overview and campaign development process. PRAB: Patient Research Advisory Board.

Findings from the *EndoPhoto Study* and our team’s previous research highlight the importance of sharing evidence that validates the experiences of people affected by endometriosis, helps people feel they are not alone, fosters hope, and recognizes the strengths of those affected. Our team’s pre-existing website showcased *EndoPhoto* results via images and quotes (*EndoPhoto* website [17]). The original website was co-created by researchers, clinicians, and patient partners to disseminate information and resources related to endometriosis. As guided by our Patient Research Advisory Board (PRAB; a group of people with lived experience of endometriosis), we chose to disseminate the *EndoPhoto Study* findings and *EndoPhoto* website to a public audience through a social media campaign. The goal of the campaign was to amplify the stories shared by Asian women regarding their experiences during the COVID-19 pandemic while focusing on disrupting silence related to the medical dismissal, social isolation, and cultural stigma of pelvic pain and endometriosis. As such, we recognized the relevance of using a trauma-informed approach to develop and implement the campaign. See Figure 1 for a project overview and the campaign development process.

### Objective

Our primary objective is to provide information on the steps we took when developing a social media campaign informed by the principles of a trauma-informed approach. Our secondary objective is to share the engagement results of the social media campaign. The target audience includes individuals and teams interested in trauma-informed social media campaigns, particularly those disseminating health-adjacent research findings. We begin by sharing information about our team, followed by information on trauma-informed approaches and social media dissemination. Lastly, we share the four steps that can be taken when developing a trauma-informed social media campaign: (1) frame the campaign, (2) create content and manage the campaign, (3) measure campaign impact, and (4) conduct postcampaign reflections.

### Our Team

We are a multigenerational team with diverse genders, sexualities, and ethnicities, and are committed to improving the understanding and awareness of endometriosis through cutting-edge interdisciplinary research and knowledge translation. We recognize the importance of disseminating intentionally curated, evidence-based, and nuanced research findings to the endometriosis community and the public. Our team includes researchers, clinicians, health care trainees, and patient partners who are part of our PRAB. We are affiliated with the Endometriosis and Pelvic Pain Laboratory at the University of British Columbia, Canada.

### What Are Trauma-Informed Approaches?

The formal conceptualization of trauma-informed care was first introduced by Harris and Fallot [18] in the context of mental health and substance use treatment systems. However, these principles have long-standing roots in community-based practices, including those within Indigenous traditions [19]. Since its inception, trauma-informed care has been adapted to various disciplines, with the framework of the Substance Abuse and Mental Health Services Administration (SAMHSA) often cited [20]. Trauma-informed approaches often acknowledge that trauma is widespread, and they actively support creating systems that promote physical and psychological safety [20].

SAMHSA defines trauma broadly, encompassing experiences at individual and structural levels that can be considered emotionally harmful [20]. SAMHSA’s trauma-informed approach rests on four key assumptions: (1) realizing that trauma is widespread and can deeply affect individuals, communities, and societies; (2) recognizing the signs of trauma; (3) responding to trauma by integrating trauma-informed approaches; and (4) resisting retraumatization [20]. These assumptions are operationalized through six guiding principles: (1) safety; (2) trustworthiness and transparency; (3) peer support; (4) collaboration; (5) empowerment, voice, and choice; and (6) cultural, historical, and gender issues [20].

In health care settings, trauma-informed approaches in the provision of care have been shown to improve negative mental health symptoms and increase patient satisfaction, especially among populations with histories of trauma or medical dismissal [21]. Trauma-informed approaches are particularly relevant in sexual and reproductive health, where patients are frequently asked to disclose sensitive information and are more likely to have experienced prior health care–related trauma [22]. Trauma-informed approaches are relevant for people with endometriosis as they have described feelings of shame and emotional distress related to their health care encounters where their symptoms have been diminished, normalized, or dismissed [23]. Interactions with health care systems and providers—as well as with broader public discourses that minimize people’s experiences of endometriosis—have been further characterized as harmful, disempowering, and socially isolating. These experiences highlight the importance of using a trauma-informed approach that prioritizes safety, empowerment, and collaboration [23].

### Social Media, Knowledge Dissemination, and Trauma-Informed Approaches

*Social media* is broadly defined as a digital space centered around information sharing and human connection [24]. Social media has increasingly become a pervasive aspect of everyday life and a powerful knowledge dissemination tool where various social media platforms, such as Facebook and TikTok, have been used by the health care community for patient education, peer support, and advocacy [25-27]. Social media platforms are participatory and easy to access, allowing the dissemination of information and rapid engagement of large, globally-connected audiences [28]. Content shared on social media platforms can provide unique opportunities to build a community, share experiences, and influence public health discourse [26].

There are also challenges with social media. For example, the nature of instant access to information and a lack of fact-verifying measures can lead to the unchecked and rapid spread of misinformation and disinformation to the public. Moreover, algorithms may incidentally lead to retraumatization and feelings of stigmatization [29-31]. Furthermore, considering people often consume social media content in isolation, it is difficult for content creators to recognize potential traumatization or retraumatization of viewers, highlighting the importance of intentionally creating and sharing content.

As knowledge dissemination of health-adjacent information increasingly moves onto social media platforms, trauma-informed approaches appear particularly relevant and potentially useful when working in these virtual environments. While there is limited guidance for applying trauma-informed approaches in digital spaces [32], the literature is emerging. We drew upon 3 frameworks that highlighted the potential of these approaches to reduce harm when sharing health-adjacent information digitally.

First, Josephs et al [33] emphasized 3 key pillars for digital trauma-informed design specific to sexual and reproductive health: privacy and confidentiality, intuitive and representative designs, and inclusive language. Second, *trauma-informed computing*, introduced by Chen et al [34], presents a framework guiding the adaptation of trauma-informed principles to digital design. This framework recognizes that digital tools can cause or exacerbate trauma and seeks to enable safer technological experiences [34]. Key adaptations of trauma-informed principles for online settings include safety, trustworthiness, peer support and collaboration, empowerment and choice, and cultural sensitivity.

Lastly, Scott et al [30] built upon the framework from Chen et al [34], adding specific aspects and examples to consider when applying trauma-informed approaches to social media engagement. The framework from Scott et al [30] outlines six guiding principles: (1) safety (eg, safe data collection and storage, and relaxing colors); (2) trustworthiness and transparency (eg, transparent about what user data are collected and why); (3) peer support (eg, protection for those sharing their unique stories); (4) collaboration and mutuality (eg, co-design with people having lived experience); (5) empowerment, voice, and choice (eg, no real names); and (6) cultural and historical gender issues (eg, acknowledge algorithmic biases).

## Developing Our Trauma-Informed Social Media Campaign

### Step 1: Frame the Campaign

#### Determine Relevant Trauma-Informed Principles

Based on the previously mentioned frameworks, we adapted our campaign to focus on the following principles of a trauma-informed approach: (1) support and collaboration, (2) trustworthiness and transparency, (3) safety, (4) empowerment and voice, and (5) cultural and gender sensitivity. These principles were used throughout the campaign development process. Table 1 provides an overview of how we approached incorporating trauma-informed principles throughout the campaign.

**Table 1.** Overview of our approach.

Trauma-informed guiding principle	Our approach and examples	Considerations
Support and collaboration	Step 1: Frame the campaign Co-designed with PRAB^a^ members and a diverse interdisciplinary team of experts Incorporated feedback throughout the process Shared content from lived experiences Engaged with influencers we had a previous relationship with to promote content Step 4: Conduct postcampaign reflections Reflected as a team on successes, challenges, and learnings for future social media engagement	More time may be needed to include the feedback and ideas of all team members, and thus, there may be a longer timeline to project completion
Trustworthiness and transparency	Step 1: Frame the campaign Informed participants of the purpose/content when consenting to the original study Obtained explicit and ongoing consent related to the use of data Step 2: Create content and manage the campaign Created content highlighting our research team’s positionality statement Created content highlighting transparency around funding and what this meant Step 3: Measure campaign impact Intentionally collected metrics that were not overly invasive Intentionally collected metrics that were inclusive of multiple ways of engaging with content, recognizing that people may engage with content differently	Viewers may have personal negative feelings about funders Additional human resources are needed to gather confirmatory consent from participants Opt-out versus opt-in could incidentally include photos that participants did not want to share, but they did not see the email
Safety	Step 1: Frame the campaign Engaged with specific platforms (Instagram and Pinterest) Step 2: Create content and manage the campaign Created content using gentle, muted color palettes in content creation (eg, using light yellow) Created content in grouped images in collage format and shared select images Created content with deidentified images by blurring faces or including images where people were masked Moderated comments Included content warnings regarding sensitive topics	Participants may provide images that violate design principles and gentle colors Additional human resources are required for moderating comments Not including all experiences captured, as many images were not selected Not as wide a reach due to including only select friendly platforms Temporary stories reduce reach Deidentifying images or using the photos without the original captions may alter the intended goal and impact of the image, limiting participant creativity
Empowerment and voice	Step 1: Frame the campaign Leveraged platforms to amplify voices Step 2: Create content and manage the campaign Created content that used friendly, nonstigmatizing, and everyday language when captioning photos Made changes to the content based on algorithm limitations while staying grounded in authentic patient voice and experiences	Potential for less reach when only using select platforms, and the reach to certain groups (eg, older people who more often use Facebook) might be reduced; demographics can vary across platforms Participant caption is not always included with a photo, which could change the intended meaning Focus on “positive” aspects due to the algorithm could represent a one-sided or skewed representation of experiences Sharing content that is “trauma-informed” may incidentally portray the information and experiences as being neutral or positive
Cultural and gender sensitivity	Step 2: Create content and manage the campaign Created content that ensured sharing only “positive” language and experiences due to the Meta algorithm Created content that avoided stereotypes and stigmatization Created content that avoided hypergendered content	Excluding images that depict too much pain or have identifying information may reduce the transparency of people’s experiences Some people might feel that the nonhypergendered colors and content do not relate to them as much

a PRAB: Patient Research Advisory Board.

#### Identify a Theoretical Approach

Theoretical approaches provide the foundation for a research study, offering structure and guidance when developing objectives, methods, and analysis [35]. Throughout the development of the campaign, including content creation, data interpretation, and dissemination strategies, we were primarily guided by the principles of intersectional feminism and integrated knowledge translation (IKT). An intersectional feminist perspective informed our understanding of how overlapping identities, such as race, gender, and country of origin, shape individuals’ health care experiences. IKT is a collaborative process that emphasizes partnership between researchers and knowledge users throughout all stages of research [36]. IKT informed how we identified priorities, designed methods, interpreted data, and shared results [36]. Unlike traditional models that position researchers as the primary producers of knowledge, IKT recognizes the expertise of both researchers and community partners, aiming to minimize power differentials and promote equitable, contextually relevant knowledge creation [37].

#### Identify Campaign Goals and Messaging

Our main goal for the social media campaign was to share key research findings from the *EndoPhoto Study,* increase awareness of Asian women’s experiences living with endometriosis, and direct viewers to our newly developed interactive *EndoPhoto* website [17]. In conducting the campaign, a second goal was to foster a sense of validation, emotional safety, and support for both previous research participants and audiences, and minimize their risk of retraumatization.

The campaign’s central message—*your pain is real, you are believed, and you are not alone*—matched the overall messaging of content produced by our team and shared on the website. Additionally, this message was consistently emphasized across all digital platforms. The team deliberately declined to create a new hashtag for the campaign, given the objective of using social media as a mechanism to reach a wide audience of users rather than share isolated content with limited reach. As such, all the posts incorporated the widely recognized community hashtag #ThisIsEndo, supplemented by topic-relevant hashtags.

#### Determine When and Where to Launch the Campaign

We launched the campaign in March 2025 to coincide with Endometriosis Awareness Month. The Endometriosis and Pelvic Pain Laboratory had a previously established Instagram profile (@pelvicpainendo) with approximately 1000 followers before the campaign and had a Pinterest account (@pelvicpainendo), which was created for this and future campaigns. These platforms aligned with the campaign’s visual and trauma-informed goals, offering features such as content warnings and comment moderation. The content shared was similar for both platforms but adapted in format to best use each platform’s features. For example, Instagram’s reels, stories, and carousels supported a balance of educational content and personal narratives, while Pinterest enabled thematic curation through boards and infographics. Pinterest has a different set of users and is more aligned with artistic communities.

Considering that Pinterest policies restrict the use of paid advertisements for new accounts, a soft launch on the platform began on February 19, 2025. Because of this, we published several posts prior to the full campaign, which allowed us to generate early interest, establish baseline engagement, and be considered an established account.

### Step 2: Create Content and Manage the Campaign

#### Overview

The content that was shared during the social media campaign was initially designed by HD (quote posts), GJY (image posts), and WZ (videos/reels), who drew upon findings from the *EndoPhoto Study* and website content under the guidance of the announcement post creator and content lead (HN). Before final approval, all content was reviewed and discussed with the broader research team during team meetings every 2 weeks and PRAB meetings every 2 months. In total, the campaign featured 41 posts between March 1 and March 31, 2025, across Instagram and Pinterest. In this paper, we chose to share the images of content only from Instagram owing to the easy viewability of the entire post. In the following section, we discuss how we enacted these aspects in content creation and when managing the campaign. It is important to note that although these aspects are presented as being separate ideas, many have overlapping and related actions.

#### Incorporating Support and Collaboration

We defined support and collaboration as intentional actions taken to meaningfully engage with those having lived experience and others in the community doing similar work. As such, PRAB members defined campaign goals; reviewed and co-developed content; and ensured that materials were safe, empowering, and contextually relevant. The PRAB involvement created an opportunity for safety by foregrounding lived experience and ensuring that social media content reflected their values and perspectives. The campaign was also managed by an external communications agency, where the partner and social media specialist (SL) was someone with lived experience of endometriosis who simultaneously acted as a member of the PRAB.

We also took a collaborative approach to promoting the campaign, leveraging our known and existing networks. This involved approaching familiar social media accounts, including science communicators, nonprofit organizations, news outlets, and independent influencers, and asking them to share and promote our content. The campaign also partnered with advocacy organizations, including The Endometriosis Network Canada, to broaden outreach and ensure alignment with existing efforts in the endometriosis advocacy landscape.

#### Incorporating Trustworthiness and Transparency

**Figure 2.**
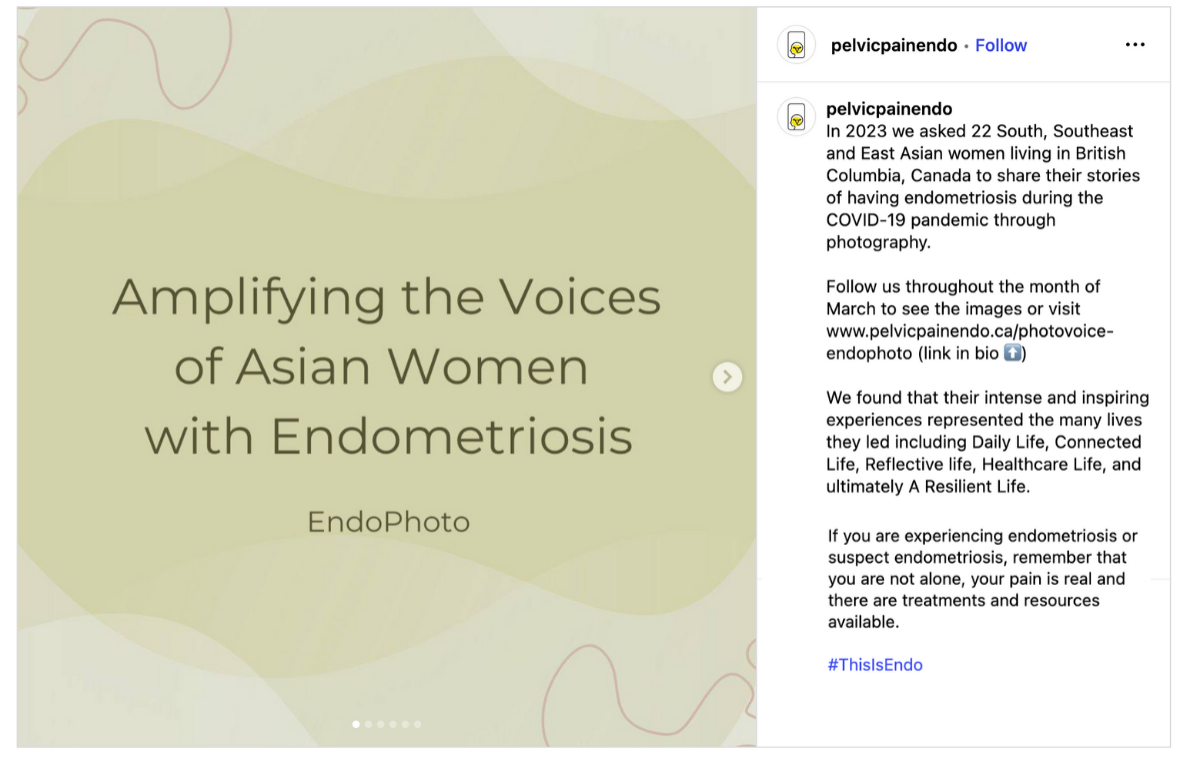
Example of an announcement-style post on Instagram.

When creating content, we considered trustworthiness and transparency aspects that involved disclosing who is behind the campaign, our goals, the funders, and where the content came from through “announcement” posts (Figure 2). Prior to the campaign, we were transparent with the participants of the *EndoPhoto Study* about how we were using their data.

All participants in the *EndoPhoto Study* provided explicit informed consent to have their photos used in a social media campaign. We also provided participants with a lay summary of the results via email that included an “action required” message, showed participants the website [17] where photos had been included in a virtual gallery, and gave participants the ability to withdraw their photos and quotes at any point. We also strongly encouraged *EndoPhoto Study* participants to review their photos to ensure they were comfortable with these being shared. No participants opted out of their photos being shared.

#### Incorporating Safety

**Figure 3.**
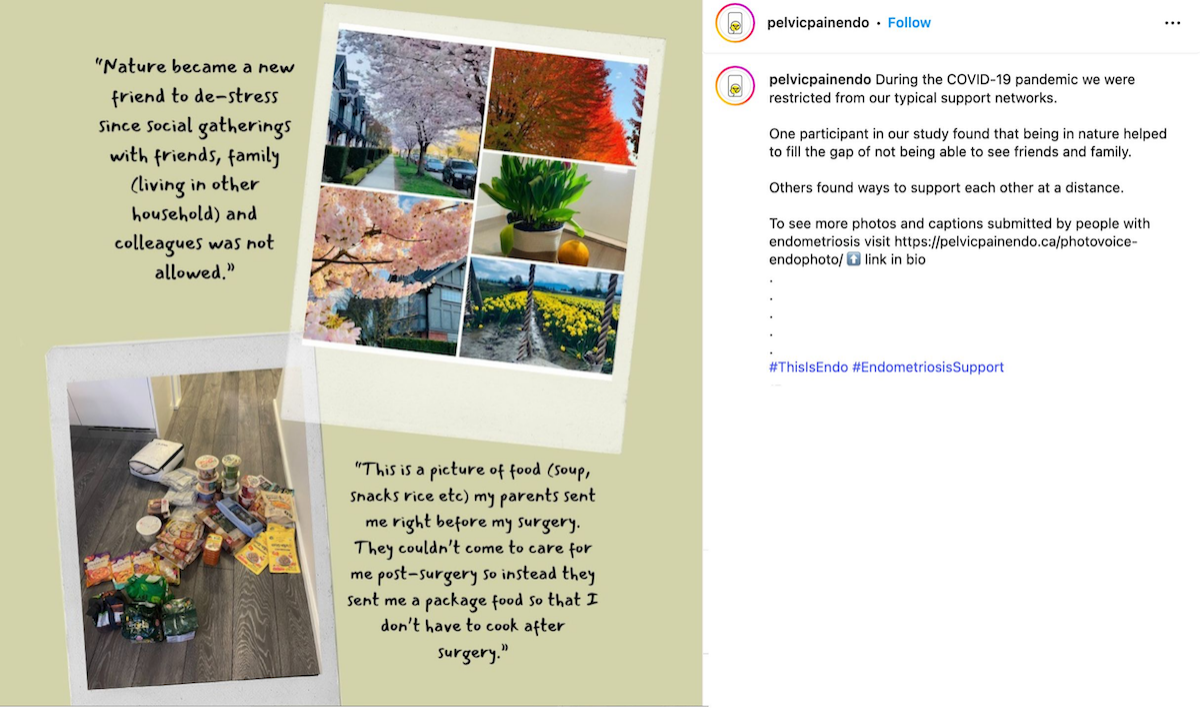
Example of a collage of nature and social support.

When considering safety, we focused on the principles of privacy and confidentiality from Josephs et al [33], and prioritized safety and preventing retraumatization of participants of the *EndoPhoto Study* whose images we shared. First, when creating content, to maintain the emotional safety of those who participated in the research and shared their photos, we intentionally curated visual content in a way that still honored participants’ lived experiences. We did this by choosing images that were not intimately personal or overtly medical in nature, or that did not depict individual people in moments of visible distress. Instead, the campaign showcased strength-based visuals, such as nature scenes, symbolic objects, and comforting moments like participants’ pets offering support (Figures3  4).

**Figure 4.**
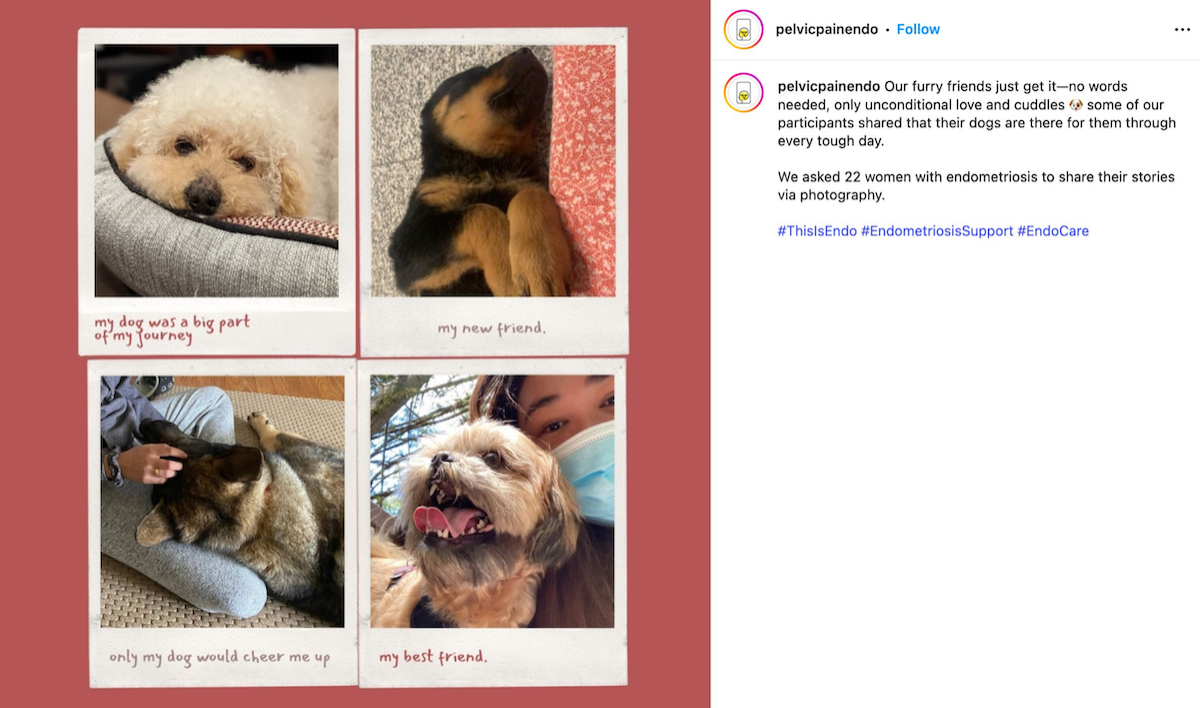
Example of a post about pets providing comfort.

When possible, in content creation, images were paired with participant-authored captions that emphasized resilience, healing, and other personally meaningful themes to balance narrative authenticity with emotional safety. We also intentionally blurred faces to protect identities or selected images that further supported anonymity, such as those in which individuals used a surgical mask (Figures4  5).

**Figure 5.**
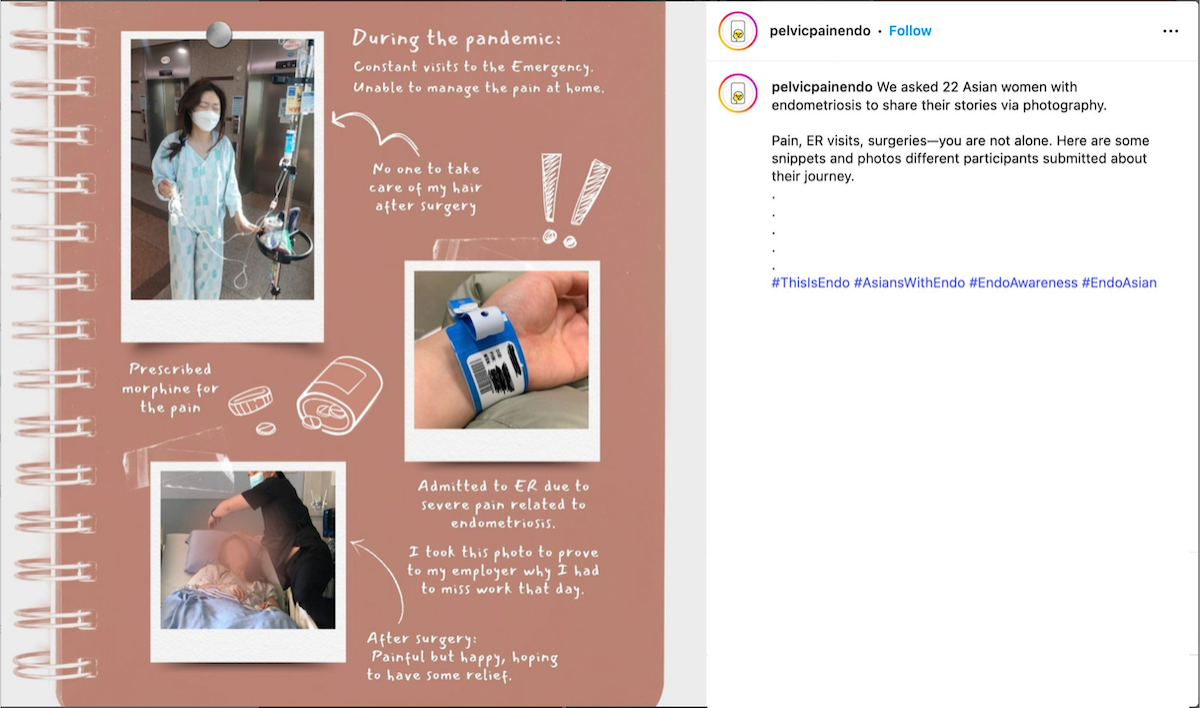
Example of identity protection on Instagram.

**Figure 6.**
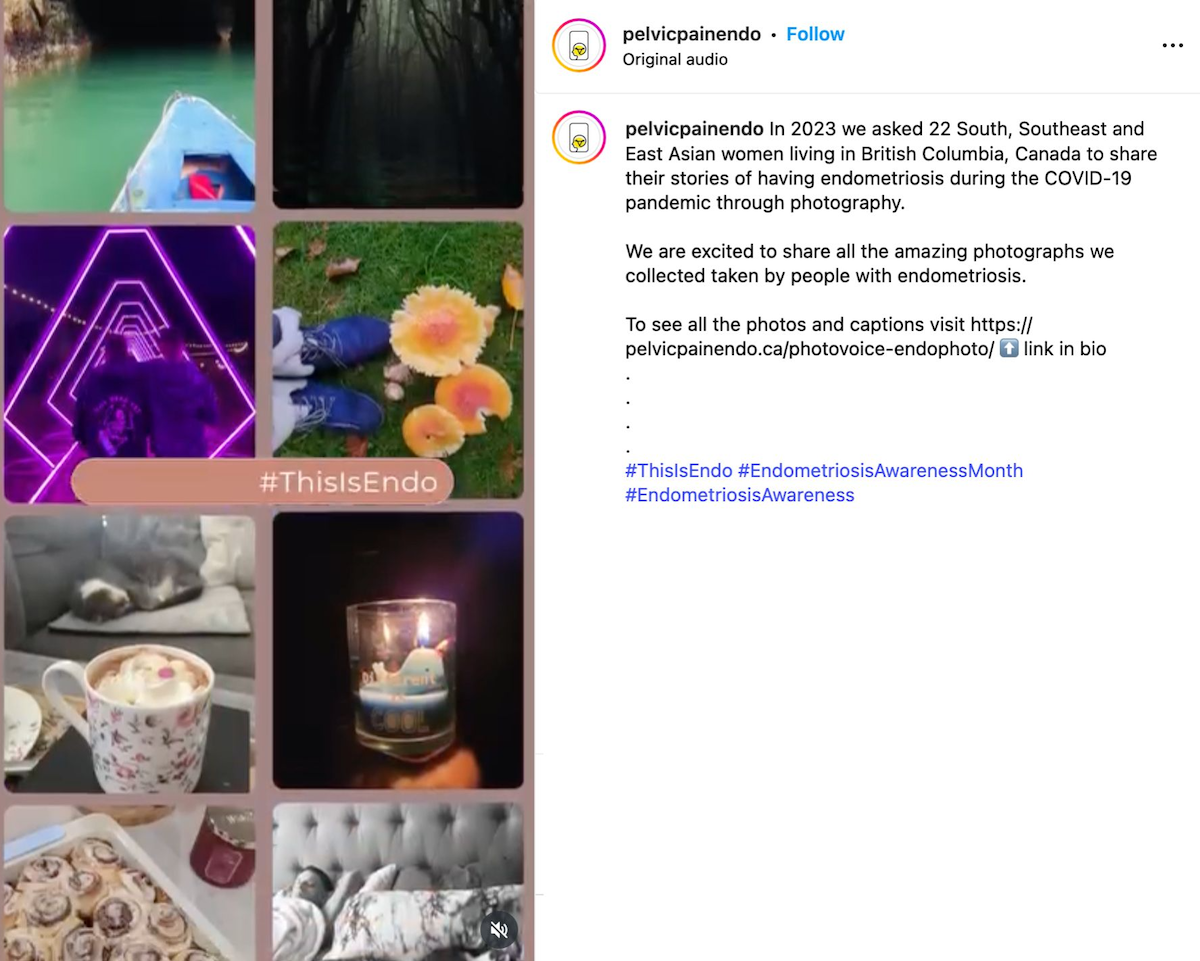
Example of a shared collage of participant photos from an Instagram reel.

Second, when creating content, careful consideration was given to how participant photos were shared. We opted to present images as collages or grouped images rather than posting them individually (Figure 6). This approach was chosen to minimize the risk of certain photos receiving disproportionately more “likes” or “shares” than others, which could cause distress among some participants if they noticed their photos were less “liked.”

Third, based on recommendations from our social media specialist and patient partners, Instagram and Pinterest were specifically chosen as platforms for this campaign, given that they are well-suited to image-based storytelling and, anecdotally, were considered less volatile during the dates of the campaign, with lower rates of reproductive health online harassment compared with other well-known platforms.

Fourth, elements of safety were further considered through active moderation of comments to identify and remove hate speech, trolling, and unsolicited medical advice; however, we did not find that these were issues in this campaign.

#### Incorporating Empowerment and Voice

**Figure 7.**
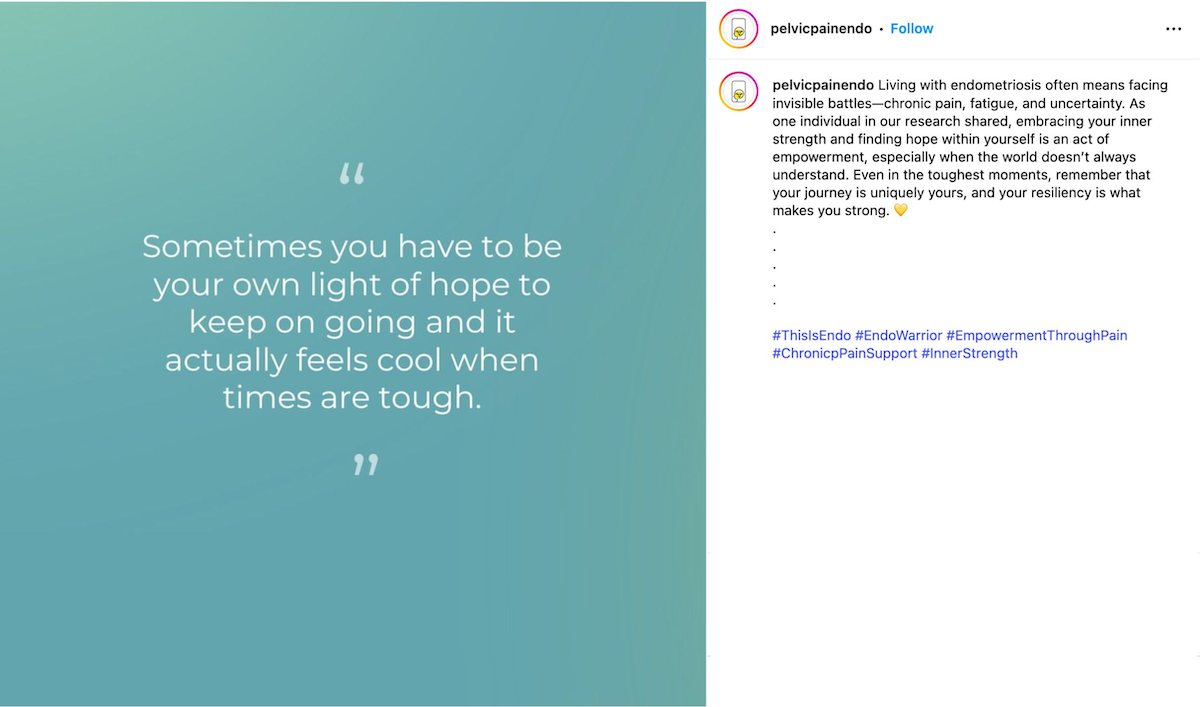
Example of quote-based content on strength while living with endometriosis.

We considered empowerment and voice to focus on ensuring that we were truthfully representing participant experiences of the *EndoPhoto Study*, opting to create content that was more strength-based and showcased resilience and empowerment while also sharing the reality of experiencing endometriosis (Figures7  8). In order to accomplish this, we created content that used nonstigmatizing, everyday language and often incorporated participants’ own words in explaining the context of the photos (Figure 9).

**Figure 8.**
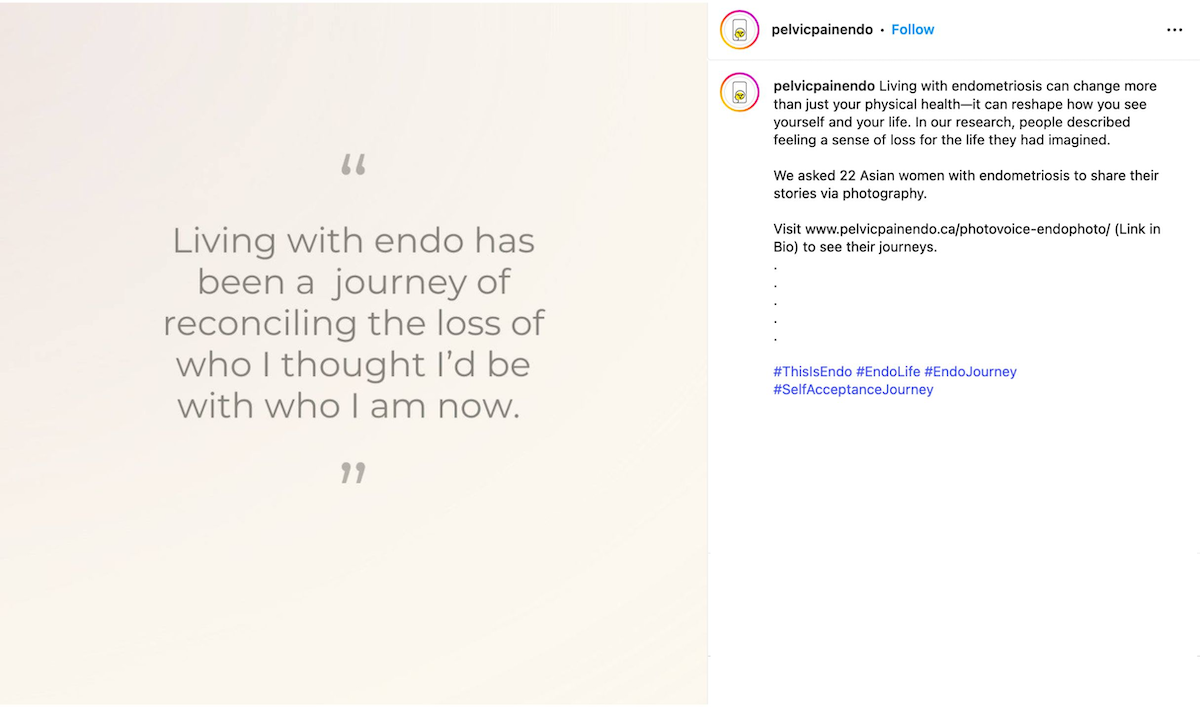
Example of quote-based content on living with endometriosis.

**Figure 9.**
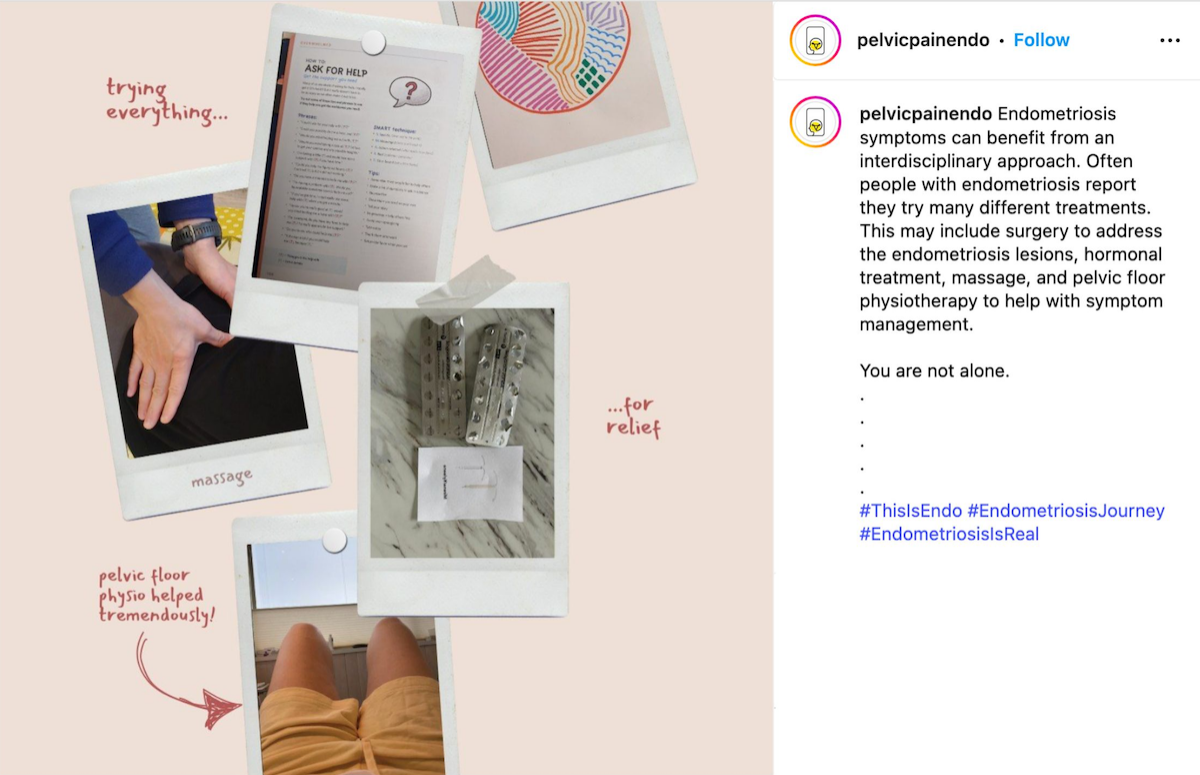
Example of everyday language use in a caption.

**Figure 10.**
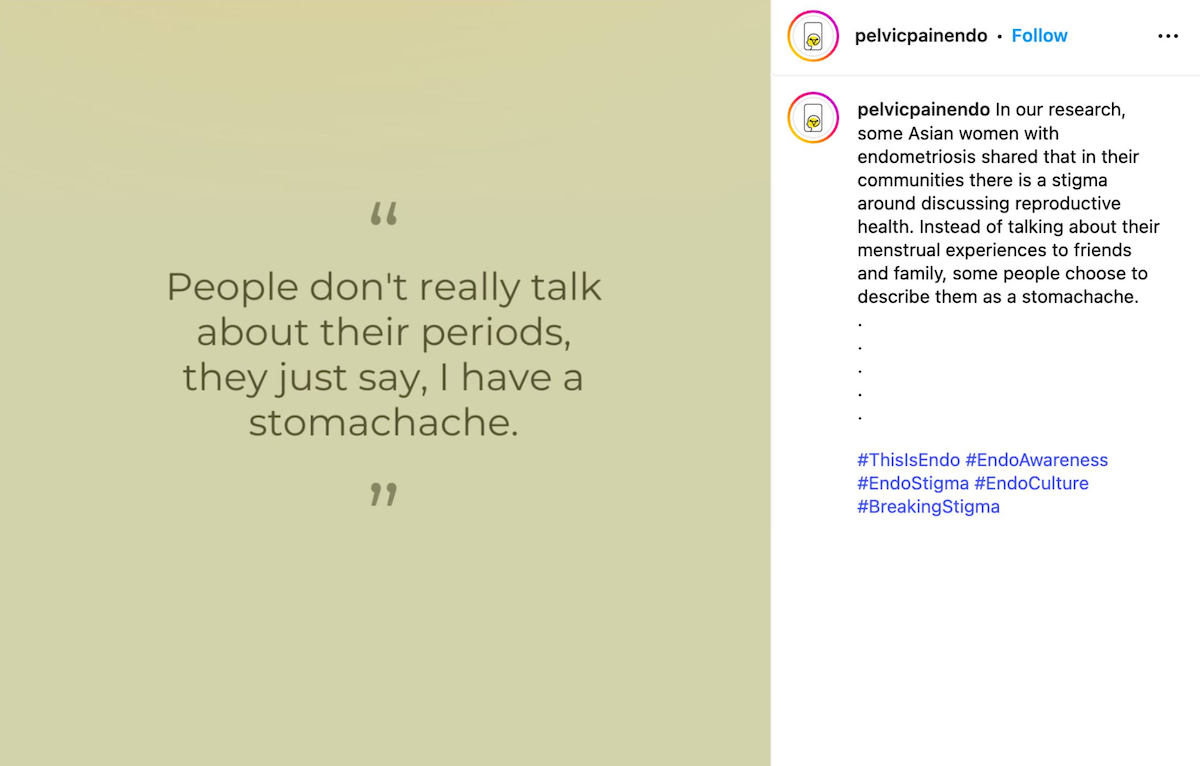
Example of content highlighting cultural taboos around menstruation.

By integrating visual narratives with participant-authored captions, the campaign created opportunities for individuals to reclaim agency in narrating their health care experiences, particularly where medical and workplace systems had previously been invalidating. For example, 1 post focused on how cultural taboos surrounding menstruation can lead to a lack of communication and discussion of pain. This caption drew attention to the compounding effects of stress, isolation, and reduced access to care (Figure 10). Together, we intended these posts to help humanize the lived realities of people with endometriosis while fostering empathy, reducing stigma, and encouraging public dialogue.

#### Incorporating Cultural and Gender Sensitivity

**Figure 11.**
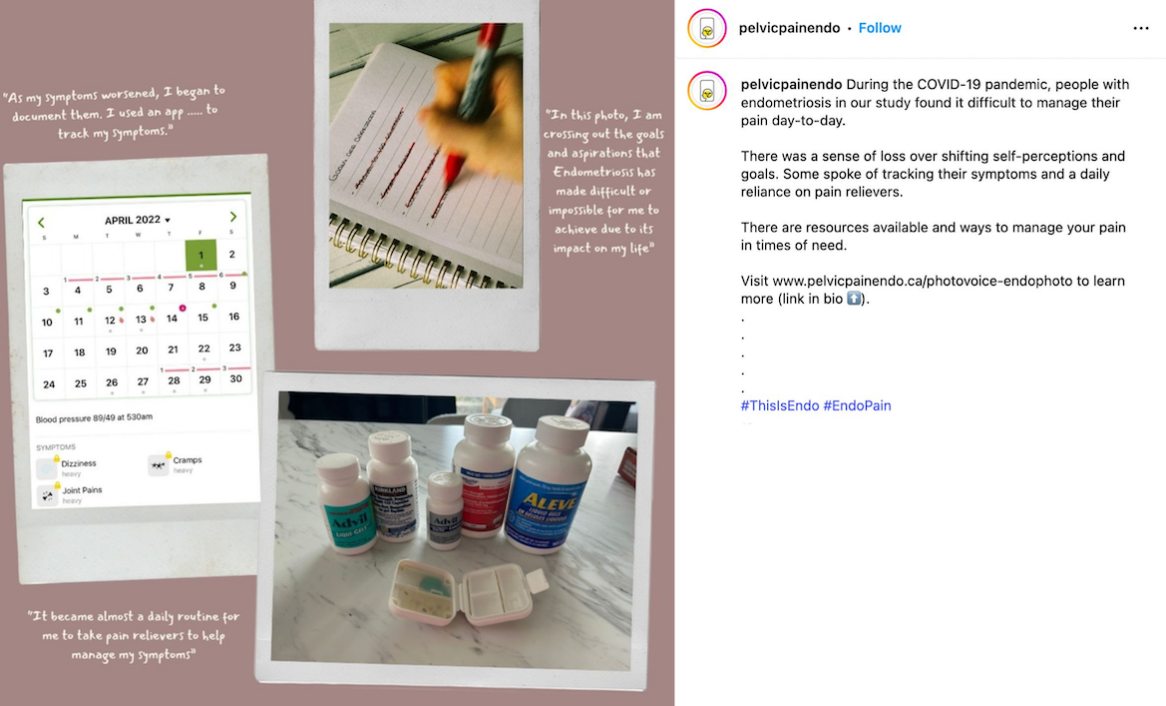
Example of the avoidance of hypergendered or stigmatizing language or colors.

Striving for cultural and gender sensitivity, we carefully curated content to avoid sensationalism, incidental stigmatization, stereotypes, clinical or diagnostic language, and potentially distressing imagery. The campaign content aimed to disrupt the silence surrounding pelvic pain and endometriosis, particularly the effects of medical dismissal, social isolation, and cultural stigma. Considering the gendered nature of endometriosis—and although all the participants whose photos we shared identified as cisgender women—we intentionally avoided making the content hyperfeminized or gendered toward women exclusively. We also aimed to avoid perpetuating stereotypes and hyperfeminized content by choosing a color palette that was intentionally calming while not overly gendered (Figure 11).

### Step 3: Measure Campaign Impact

We used platform-integrated analytics (Instagram Insights and Pinterest Analytics) to monitor primary performance indicators. The metrics included reach, engagement (likes, shares, comments, profile visits, and link clicks), and website page visits (for definitions, see Table 2). We reviewed these metrics throughout the campaign to enable real-time optimization of posting frequency and timing, as well as advertising spend based on platform recommendations and observed audience behavior. Over the 31-day campaign, the website attracted 6326 unique users (for additional engagement measures, see Table 3).

**Table 2.** Consolidated definitions of terms used to describe campaign engagement.

Term	Definition
Reach	Reach is defined as the number of unique users who have seen the online content at least once
Engagement	Likes, shares, comments, profile visits, and link clicks
Impressions	The total number of times the content is presented to potential users on a screen
Volume of sessions	Number of visits to the website
Dwell time	The duration of time people view the content
Click-through rate	The percentage of people who click on a link to the website within the content

**Table 3.** Engagement results from Instagram and Pinterest.

Platform and metric		Value
Overall		
Total social impressions, n		8,540,528
Instagram		
Engagement^a^, %		6.23
Advertisement impressions, n		7,941,457
Advertisement reach, n		3,550,309
Pinterest		
Total impressions, n		581,081
Total engagement, n		5528

a With regard to engagement, on Instagram, anything above 6% is considered high engagement, and on Pinterest, anything between 1% and 2% is considered average engagement [38,39].

**Figure 12.**
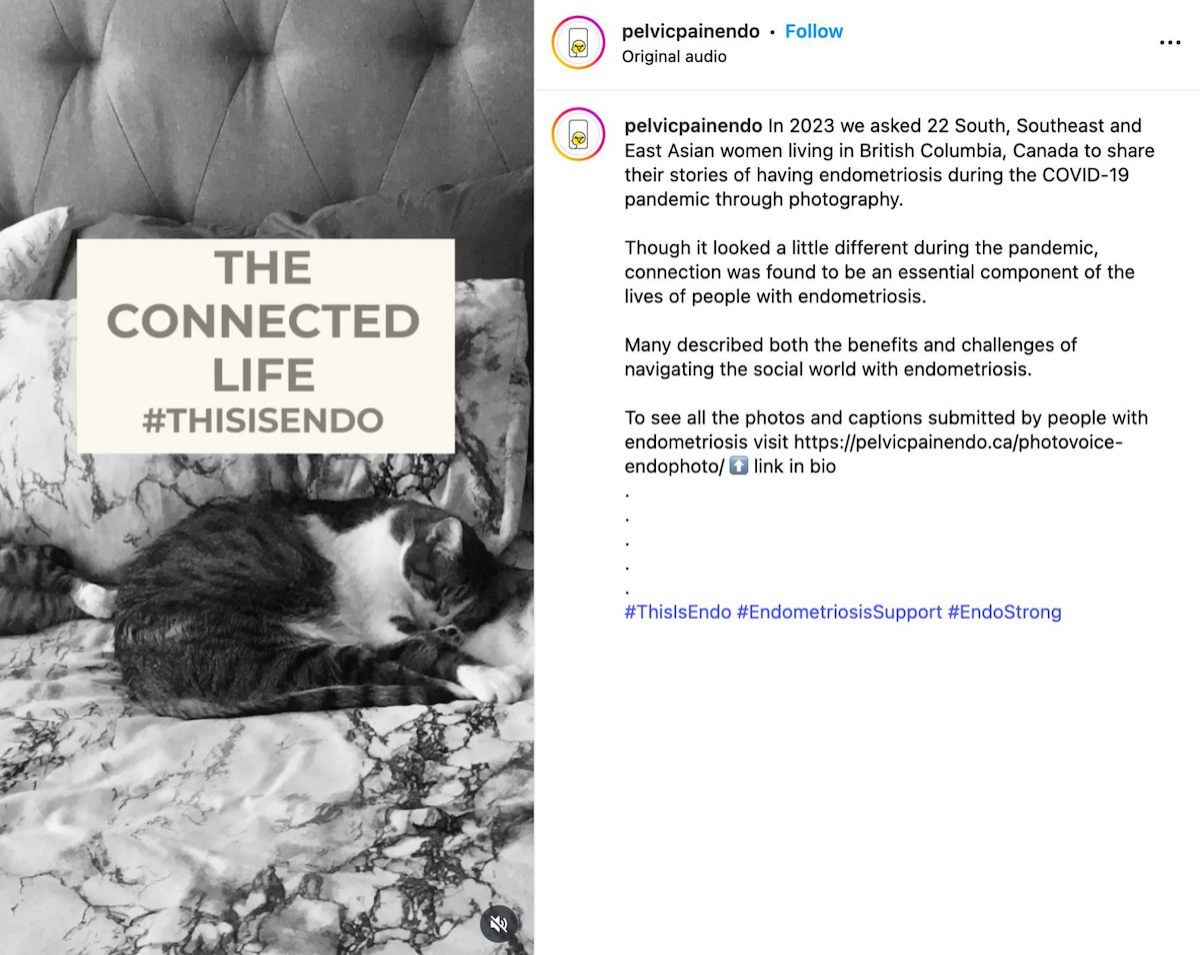
Still image from a reel that “went viral” during the campaign.

**Figure 13.**
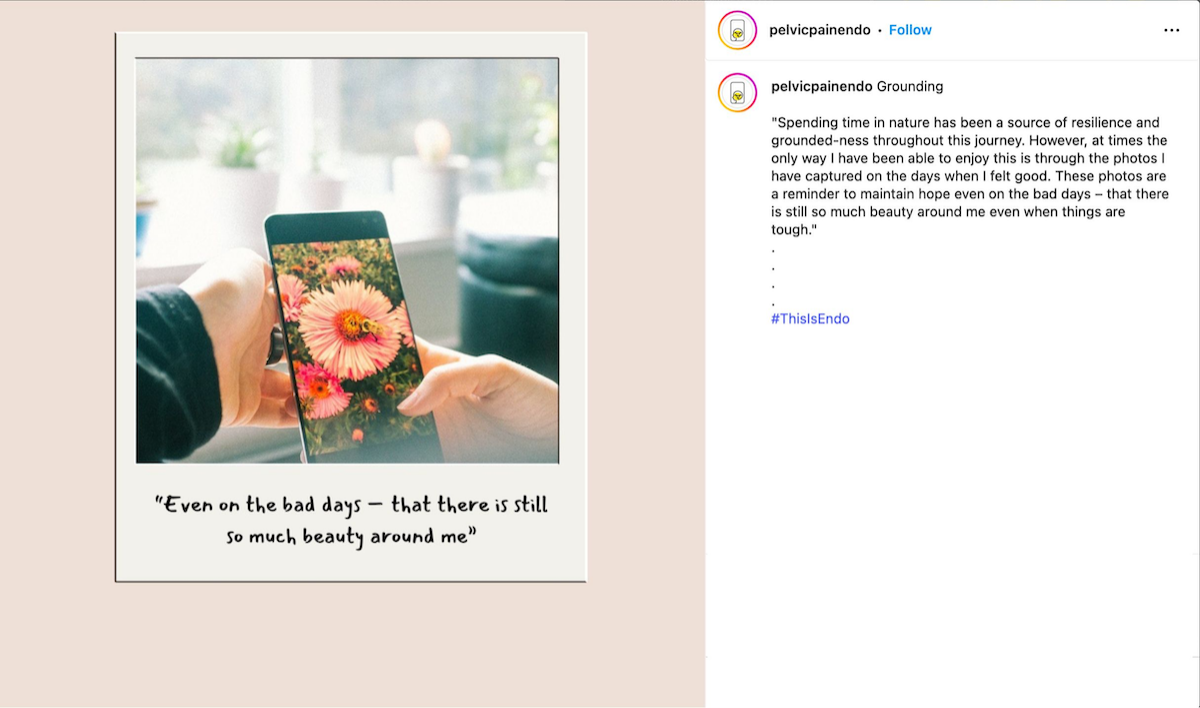
A post that “went viral” during the campaign.

**Figure 14.**
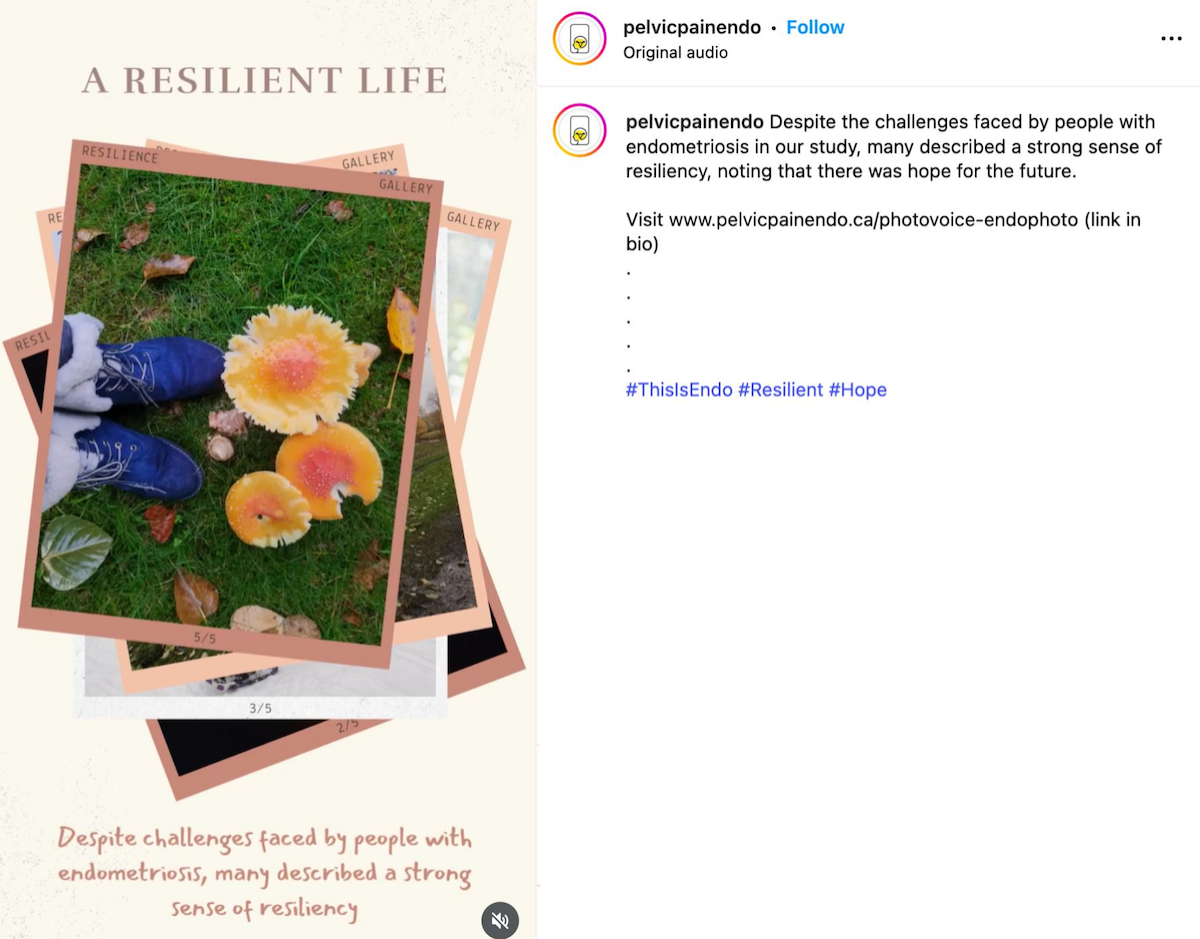
Still image taken from the second reel that “went viral” during the campaign.

Instagram generated both the greatest volume of sessions and the longest dwell time. Pinterest’s shorter dwell time likely reflected the account’s infancy (new profile, first campaign, and algorithmic learning period). The high number of impressions was largely attributed to 3 posts that “went viral” during the campaign, meaning they received over 1 million views each (Figures 12-14). These posts reflected messages of support, resiliency, and healing. Advertisements drew in the largest number of platform users to the page, accounting for most of the sessions and engagement, with arguably the least human resources.

### Step 4: Conduct Postcampaign Reflections

#### Identify What Went Well

To increase the reach of the social media campaign and the number of people seeing the online content at least once, we paid for advertisements on both platforms, with a total budget of CAD $3000 (US $2160). We found that advertisements required few human resources and were a relatively inexpensive way to increase reach. Posts that were advertised resulted in greater reach than posts that were not advertised. For our paid advertisements, a detailed audience profile was developed to guide content creation and advertisement targeting. The intended audience included individuals who either had a confirmed or suspected endometriosis diagnosis, were assigned female at birth, were of reproductive age (inclusive of all gender identities, sexual orientations, and relationship statuses), had moderate levels of health literacy, and understood English. We did not explicitly target Asian women and people living with endometriosis, as race and disease data are not captured for users by social media platforms explicitly. High-frequency search terms related to pelvic pain, endometriosis, and Asian experiences were identified to optimize advertisement discoverability (eg, pain, symptoms, self-care, journey, resilience, and reflection).

One of the campaign’s primary strengths was its collaborative, interdisciplinary approach. The involvement of patient partners through the PRAB ensured content authenticity and emotional safety. In addition, working with a social media specialist was invaluable for navigating the landscape of digital media. The campaign also benefited from sufficient funding, allowing strategic investment in high-performing advertisement formats and continuous optimization based on analytics.

Our team’s expertise, including content development led by those with lived, clinical, and research experience, added legitimacy, credibility, and multiple perspectives that countered misinformation in digital health spaces. Importantly, the campaign filled a representational void by centering the narratives of South, East, and Southeast Asian women with endometriosis, a demographic historically underrepresented in both research and advocacy.

#### Identify Challenges

**Figure 15.**
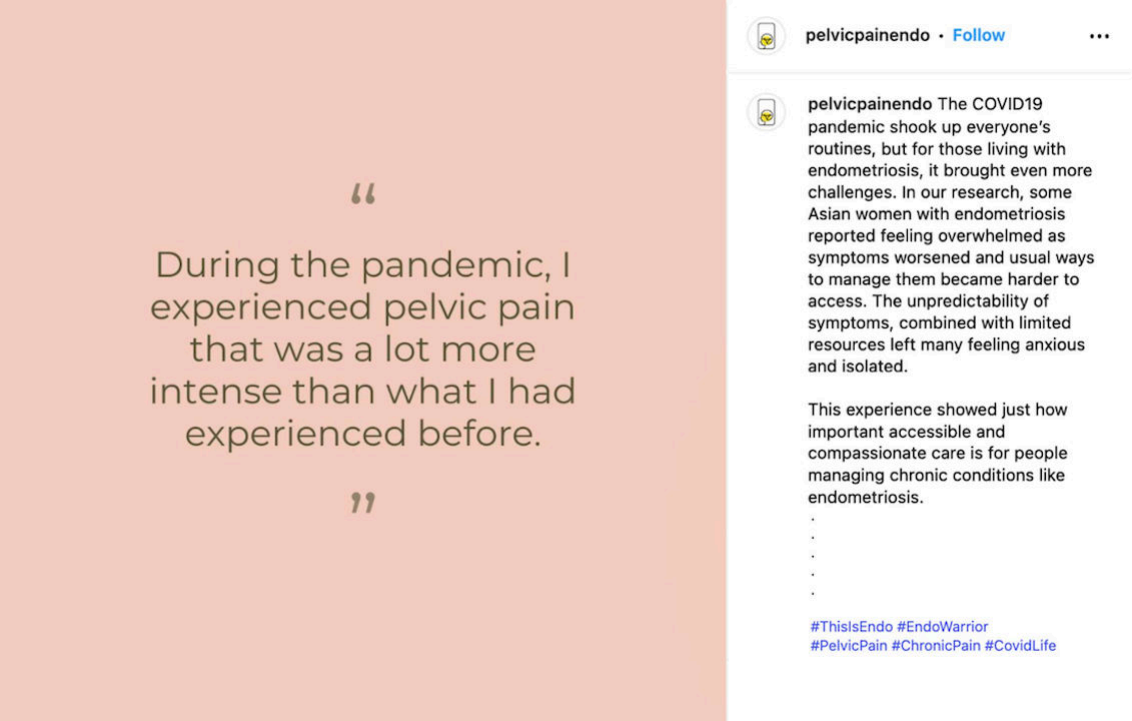
An original post on Instagram that was flagged by the algorithm as being “negative”.

Early in the campaign (March 3, 2025), our Instagram account was flagged by the Meta algorithm and included in a category called “Health and Wellness” owing to a post that was identified as being “negative” (Figure 15). This category was designed by Meta to reduce “negative” advertising and monetization of organizations that use advertising to sell products and services. Although we were not advertising products, our placement in this category limited what content could be promoted. Posts that included any features flagged as “negative” by the algorithm were prohibited from being advertised. Considering that endometriosis experiences often involve challenges, our preplanned content required significant changes in order to meet the algorithm’s criteria.

## Discussion

### Lessons Learned

This manuscript describes the development of a trauma-informed social media campaign designed to disseminate findings from the *EndoPhoto Study*, which explored the experiences of South, East, and Southeast Asian women living with endometriosis in Canada during the COVID-19 pandemic. We intend for this work to serve as a guide for others seeking to share their research findings through social media, with broader applicability for those interested in trauma-informed campaign development. By integrating trauma-informed principles, the campaign not only centered the voices of underrepresented communities but also demonstrated the potential for digital platforms to promote trauma-informed knowledge dissemination.

Upon reviewing our engagement metrics, we found that images with quote-based content (as opposed to image- or quote-only–based content) produced the highest click-through rates on both platforms; announcement posts (eg, study overview) generated the greatest engagement; and Instagram advertisements, particularly images with quotes, outperformed other creative formats (Table 4). Although it is challenging to measure how the content truly impacted viewers, some of these indicators may provide insights into the meaning viewers garnered from the content. For example, the option to “save” content on Pinterest may suggest that some of the posts resonated enough for viewers to want to share or review the content at a later date, perhaps indicating a feeling of added value.

**Table 4.** Examples of posts and engagement.

Type of content and example in the manuscript (figure)		Instagram reach	Instagram impressions	Pinterest impressions
Image-based				
Figure 5		484 unique viewers	658 presentations	—^a^
Quote-based				
Figure 8		295	422	259; 96,371 presentations with advertisements
Announcement style				
Figure 2		1396 unique viewers	2361 presentations	—
Reel				
Figure 6		509 unique viewers	840 presentations	—
Advertisement				
Figure 14		1,924,605 unique viewers	2,506,671 presentations	—
Figure 12		1,044,746 unique viewers	1,099,259 presentations	—
Figure 9		1,043,201 unique viewers	1,455,368 presentations	—
Figure 13		1,007,878 unique viewers	1,559,976 presentations	—
Figure 10		—	—	81,523 presentations
Figure 3		14,172 unique viewers	16,963 presentations	23,674 presentations

a Not applicable.

At this time, we are unable to say definitively as to why some post types had higher levels of reach and impressions (outside of advertisements). To the best of our knowledge, there is no research literature that shows which specific types of social media content tend to perform better, as it is highly based on the content and target audience. Some social media blogs have suggested that reels, carousel-type posts or those that have multiple images (our announcement posts), and relatable content tend to have higher engagement [40]. Although these content categories are broad, they could indicate that working as a team that has lived knowledge, clinical knowledge, and social media knowledge proved beneficial in understanding the types of content that may be engaging for our audience.

While trauma-informed principles are increasingly recognized in clinical and community settings, their application to digital media, particularly social media, remains largely underexplored. This tutorial highlights how principles, such as collaboration, safety, trustworthiness, voice, and cultural and gender sensitivity, can be applied in online spaces to mitigate harm and increase engagement. Additionally, this project helps to address the critical gap in the representation of racialized individuals, particularly Asian women, in endometriosis advocacy and online discourse.

One unanticipated lesson learned was the suppression of women’s sexual and reproductive health information on Meta platforms. A recent report published by the Center for Intimacy Justice [41] highlighted that a bias exists on major Meta platforms, where organizations felt that their content and advertisements related to women’s sexual and reproductive health, including fertility and pelvic health, were being censored and overmoderated. Social media algorithms, driven by artificial intelligence, limited content visibility when posts included “sensitive” health-related terms or were deemed to violate vague platform policies such as Meta’s “Personal Health and Appearance” guidelines. This report aligned with our experience, as our content was flagged as not aligning with community standards owing to its “negative” nature and association with health care. This flag necessitated a shift toward resilience-focused and positively framed messaging only, which may have constrained the full scope of participant narratives.

### Limitations of the Campaign

Considering that platform selection was intentional, using only Instagram and Pinterest (chosen for their visual nature and perceived safety) may have excluded audiences who primarily engage with platforms like TikTok, X (formerly Twitter), or Facebook. Additionally, the metrics used to gauge success, such as views, impressions, and likes, offer limited insight into true impact. While some posts featuring animals gained viral traction, it is unclear whether the viral nature was due to their relevance to endometriosis or due to the important role that animals can play in people’s lives, prompting questions about whether high engagement with content truly reflected increased awareness or understanding of endometriosis specifically.

In addition, because the campaign relied on Meta-owned platforms, our content was influenced by algorithmic restrictions that often suppressed posts containing sexual or reproductive health terms. This may have unintentionally narrowed the range of experiences we were able to highlight. While the campaign focused on the voices of Asian women, our sample does not capture the full diversity of people living with endometriosis, including those with different cultural backgrounds, gender identities, or varying levels of access to digital spaces. Finally, even with trauma-informed strategies in place, sharing health-related stories online carries the risk of re-exposure or secondary trauma for some viewers, especially when content reflects their own experiences.

### Key Takeaways

The key takeaways are as follows:

- Trauma-informed principles can be adapted for digital health communication and effectively applied in social media campaigns.
- Posts that featured images with quotes, support networks (including pets), and announcements consistently maximized engagement, suggesting that these formats may be prioritized to ensure engagement.
- Early budget reallocation toward viral creative assets improved cost-efficiency.
- Paid advertisements created opportunities for ensuring wider reach and may be helpful in providing opportunities for content to be viewed by a larger audience.
- Inclusive online storytelling that prioritizes participant voice and emotional safety resonates with audiences and supports effective knowledge translation.
- Algorithmic biases targeted toward women’s sexual and reproductive health may necessitate creativity to avoid messages being flagged or framing messaging in “positive” ways. This can ensure a wider reach but highlights gender bias within social media platforms.

## Abbreviations

IKT: integrated knowledge translation
PRAB: Patient Research Advisory Board
SAMHSA: Substance Abuse and Mental Health Services Administration

## References

[1] Zondervan KT, Becker CM, Missmer SA (2020). Endometriosis. N Engl J Med.

[2] Johnson NP, Hummelshoj L, Adamson GD (2017). World Endometriosis Society consensus on the classification of endometriosis. Hum Reprod.

[3] Wahl KJ, Yong PJ, Bridge-Cook P, Allaire C, EndoAct C (2021). Endometriosis in Canada: it is time for collaboration to advance patient-oriented, evidence-based policy, care, and research. J Obstet Gynaecol Can.

[4] Singh S, Soliman AM, Rahal Y (2020). Prevalence, symptomatic burden, and diagnosis of endometriosis in Canada: cross-sectional survey of 30 000 women. J Obstet Gynaecol Can.

[5] Greene R, Stratton P, Cleary SD, Ballweg ML, Sinaii N (2009). Diagnostic experience among 4,334 women reporting surgically diagnosed endometriosis. Fertil Steril.

[6] Sims OT, Gupta J, Missmer SA, Aninye IO (2021). Stigma and endometriosis: a brief overview and recommendations to improve psychosocial well-being and diagnostic delay. Int J Environ Res Public Health.

[7] Bougie O, Nwosu I, Warshafsky C (2022). Revisiting the impact of race/ethnicity in endometriosis. Reprod Fertil.

[8] Williams C, Long AJ, Noga H (2019). East and South East Asian ethnicity and moderate-to-severe endometriosis. J Minim Invasive Gynecol.

[9] Kabani Z, Ramos-Nino ME, Ramdass P (2022). Endometriosis and COVID-19: a systematic review and meta-analysis. Int J Mol Sci.

[10] Demetriou L, Cox E, Lunde CE (2021). The global impact of COVID-19 on the care of people with endometriosis. Front Glob Womens Health.

[11] Leonardi M, Horne AW, Vincent K (2020). Self-management strategies to consider to combat endometriosis symptoms during the COVID-19 pandemic. Hum Reprod Open.

[12] Schwab R, Stewen K, Kottmann T (2022). Mental health and social support are key predictors of resilience in German women with endometriosis during the COVID-19 pandemic. J Clin Med.

[13] Leigh JP, Moss SJ, Tiifu F (2022). Lived experiences of Asian Canadians encountering discrimination during the COVID-19 pandemic: a qualitative interview study. CMAJ Open.

[14] Han CS, Oliffe JL (2016). Photovoice in mental illness research: a review and recommendations. Health (London).

[15] Marshall K, Howard AF, Marshall N, Noga H, Rojas HE, Leonova A (2025). Impacts of the COVID-19 pandemic on the mental health of asian women with endometriosis in canada: a photovoice study. SAGE Women’s Health (forthcoming).

[16] Marshall N, Howard AF, Marshall K (2026). Endometriosis and expressions of self-management and resilience among asian women living in canada during the COVID-19 pandemic: a photovoice study. J Public Health Res.

[17] EndoPhoto. Endometriosis and Pelvic Pain Laboratory.

[18] Harris M, Fallot RD (2001). Envisioning a trauma-informed service system: a vital paradigm shift. New Dir Ment Health Serv.

[19] Makosis P, Greenwood M (2017). What’s new is really old: trauma-informed health practices through an understanding of historic trauma. National Collaborating Centre for Indigenous Health (NCCIH).

[20] (2014). SAMHSA’s concept of trauma and guidance for a trauma-informed approach. https://www.health.ny.gov/health_care/medicaid/program/medicaid_health_homes/docs/samhsa_trauma_concept_paper.pdf.

[21] Raja S, Hasnain M, Hoersch M, Gove-Yin S, Rajagopalan C (2015). Trauma informed care in medicine: current knowledge and future research directions. Fam Community Health.

[22] (2021). Caring for patients who have experienced trauma: ACOG Committee opinion, number 825. Obstet Gynecol.

[23] Parmar G, Howard AF, Noga H (2025). Pelvic pain & endometriosis: the development of a patient-centred e-health resource for those affected by endometriosis-associated dyspareunia. BMC Med Inform Decis Mak.

[24] Burgess J, Marwick A, Poell T, Burgess J, Marwick A, Poell T (2018). The SAGE Handbook of Social Media.

[25] Davis JL, Mazzoleni G (2016). The International Encyclopedia of Political Communication.

[26] Moorhead SA, Hazlett DE, Harrison L, Carroll JK, Irwin A, Hoving C (2013). A new dimension of health care: systematic review of the uses, benefits, and limitations of social media for health communication. J Med Internet Res.

[27] Ezeilo CO, Leon N, Jajodia A, Han HR (2023). Use of social media for health advocacy for digital communities: descriptive study. JMIR Form Res.

[28] Shawky S, Kubacki K, Dietrich T, Weaven S (2019). Using social media to create engagement: a social marketing review. JSOCM.

[29] Aïmeur E, Amri S, Brassard G (2023). Fake news, disinformation and misinformation in social media: a review. Soc Netw Anal Min.

[30] Scott CF, Marcu G, Anderson RE, Newman MW, Schoenebeck S Trauma-informed social media: towards solutions for reducing and healing online harm.

[31] Abdulai AF, Howard AF, Yong PJ, Currie LM (2023). Defining destigmatizing design guidelines for use in sexual health-related digital technologies: a Delphi study. PLOS Digit Health.

[32] Zheng W, Walquist E, Datey I “It’s not what we were trying to get at, but I think maybe it should be”: learning how to do trauma-informed design with a data donation platform for online dating sexual violence.

[33] Josephs JC, Bungay V, Guta A, Gilbert M, Abdulai AF (2024). Trauma-informed technology design in digital sexual health interventions. Stud Health Technol Inform.

[34] Chen JX, McDonald A, Zou Y Trauma-informed computing: towards safer technology experiences for all.

[35] Heale R, Noble H (2019). Integration of a theoretical framework into your research study. Evid Based Nurs.

[36] Kothari A, Wathen CN (2013). A critical second look at integrated knowledge translation. Health Policy.

[37] Crosschild C, Huynh N, De Sousa I, Bawafaa E, Brown H (2021). Where is critical analysis of power and positionality in knowledge translation?. Health Res Policy Syst.

[38] What’s a good engagement rate on Pinterest?. Hudson Design Company.

[39] Polishchuk D (2022). What is a good Instagram engagement rate?. Promo Republic.

[40] Rose-Collins F (2025). Content types that perform best on Instagram. Ranktracker.

[41] (2025). The digital gag: supression of sexual and reproductive health on Meta, TikTok, Amazon and Google. https://www.intimacyjustice.org/.

